# A Synergistic Effect Based on the Combination of Melatonin with 1-Methylcyclopropene as a New Strategy to Increase Chilling Tolerance and General Quality in Zucchini Fruit

**DOI:** 10.3390/foods11182784

**Published:** 2022-09-09

**Authors:** Jorge Medina-Santamarina, María Serrano, María Celeste Ruiz-Aracil, Mihaela Iasmina Madalina Ilea, Domingo Martínez-Romero, Fabián Guillén

**Affiliations:** Postharvest Research Group of Fruit and Vegetables, Centro de Investigación e Innovación Agroalimentaria y Agroambiental (CIAGRO-UMH), University Miguel Hernández, Ctra. Beniel km. 3.2, Orihuela, 03312 Alicante, Spain

**Keywords:** chilling injury, storage, melatonin, quality, 1-MCP

## Abstract

Zucchini fruit are highly sensitive to low temperatures leading to significant peel depressions, increasing weight loss and making them impossible to be commercialized. In this study the effect on the reduction of chilling injury (CI) assaying different postharvest treatments to cv. Cronos was evaluated. We have compared the application of substances such as 1-methylcyclopropene (1-MCP) with the application of a natural origin compound as melatonin (MT), both with demonstrated activity against CI in different vegetal products. The effects of MT (1 mM) by dipping treatment of 1 h and 1-MCP (2400 ppb) have been evaluated on zucchini fruit during 15 days of storage at 4 °C plus 2 days at 20 °C. Treatments applied independently improved some fruit quality parameters in comparison with control fruit but were not able to manage CI even though they mitigated the impact on several parameters. However, when these two separated strategies were combined, zucchini cold tolerance increased with a synergic trend. This synergic effect affected in general all parameters but specially CI, being also the only lot in which zucchini fruit were most effectively preserved. This is the first evidence in which a clear positive effect on zucchini chilling tolerance has been obtained combining these two different strategies. In this sense, the combined effect of 1-MCP and MT could be a suitable tool to reach high quality standards and increasing shelf life under suboptimal temperatures.

## 1. Introduction

Zucchini (*Cucurbita pepo* spp. *pepo*), is considered a non-climacteric fruit as many others unripen vegetal fruit. The commercial harvest stage is coincident with a high fruit metabolism which deteriorates rapidly quality. On the other hand, this fruit is very sensitive to cold storage being a major problem their marketability to European countries and worldwide at suboptimal temperatures. Common postharvest strategies have been demonstrated to be effective reducing chilling injury (CI) in zucchini, as physical treatments managing temperatures [1,2,3] or relative humidity during storage [4,5] and controlling atmospheres [6,7] also through a minimal packaging [8].

Recently, chemical treatments as nitric oxide [9] or widely used treatments as 1-methylciclopropene (1-MCP) [8,10] have demonstrated a protective role delaying zucchini chilling injury. 1-MCP effectively blocks ethylene action delaying zucchini senescence and different associated physiological changes as softening and CI [10]. Strategies based into plant hormones or natural elicitors as γ-aminobutyric acid (GABA) [11], glycine betaine [12], polyamines [13,14,15] and methyl jasmonate [16] have also reduced CI impact in zucchini fruit. In this sense, melatonin has shown a positive effect delaying CI when applied as a postharvest treatment in different non-climacteric species as strawberry and pomegranate [17,18]. With respect to climacteric fruit, MT increased CI tolerance in peach [19] and tomato fruit [20] Also showing effectiveness against CI after preharvest treatments on apricot trees [21]. In these research studies the CI tolerance was associated with a major energy status obtained through the GABA-shunt pathway activation which provides extra ATP. Plant tissues demand ATP especially under metabolic stress conditions. In this sense MT, methyl jasmonate, polyamines or GABA, have been proposed as responsible of GABA shunt pathway stimulation, providing extra ATP, and decreasing ROS accumulation [17,22]. For this reason, MT treated fruit could lead to maintenance membrane permeability and balanced antioxidant system under cold stress, increasing unsaturated/saturated fatty acids ratio in both non-climacteric, and climacteric fruit [17,23].

Several of the above-mentioned strategies have been shown an additional benefit when applied in combination with MCP describing a synergistic effect delaying senescence in comparison with these technologies when applied alone. In this sense, recently, a combination of hot air treatments with 1-MCP lead to delayed softening and quality maintenance of nectarines [24]. With respect to postharvest chemical treatments, 1-MCP in combination with chlorine dioxide postharvest treatment showed a synergistic inhibitory effect on chlorophyll degradation of green pepper fruit [25]. On the other hand, dipping in calcium chloride and then applying 1-MCP was a successful strategy to improve fresh-cut strawberries and watermelon quality during storage [26,27]. Other natural origin compounds as carvacrol [28] or elicitors as methyl salicylate [29] and melatonin [30] have increased shelf-life delaying ripening when these treatments were applied in combination with 1-MCP in red pitaya, tomato fruit, and apricot respectively. However, none of these studies have evaluated the chilling tolerance provided by a combined effect of the previous mentioned strategies. For this reason, the aim of this research has been to evaluate for the first time the effect of different strategies as melatonin and 1-MCP combined or alone over zucchini CI tolerance and quality during storage at suboptimal temperatures.

## 2. Materials and Methods

### 2.1. Plant Material and Postharvest Treatments

Zucchini fruit (*Cucurbita pepo* spp. *pepo*) commercial hybrid ‘Cronos’, were harvested from a commercial greenhouse located in Orihuela (Spain) and immediately transferred to the laboratory. Fruit of uniform size (20–22 cm) were randomly divided into 3 lots of 5 homogeneous fruit for each treatment and sampling date. Control fruits were submerged in distilled water containing 0.5% Tween 20 while MT treated fruit were dipped in a 1 mM MT solution (Sigma-Aldrich, Germany >98% M5250) for 60 min. In preliminary experiments, 9 zucchini fruit were selected per MT dose and different MT concentrations (0.1, 0.5 and 1 mM) and immersion times (from 10, 30, 60, 120 and 180 min) were evaluated. These treatments were assayed alone and in combination with 2400 ppb of 1-MCP for 48 h at 12 °C following Megías et al. [10] conditions for Cronos cultivar. 1 mM MT for 1 h and combined with 1-MCP showed the best effect on reducing CI symptoms. Thus, 3 replicates of 5 zucchini fruit for each treatment and sample time were selected to repeat the experiment at the optimal conditions observed. freshly prepared MT solutions (0 and 1 mM) with 0.5% Tween 20 were used to dip the different lots for 1 h at 20 °C. Then zucchini fruit were allowed to surface dry and then all the fruit were placed in 4 different 130 L hermetic containers. One lot with MT-treated and another lot with no immersed fruit were exposed to 2400 ppbL^−1^ of 1-MCP for 48 h at 12 °C. The other two lots previously immersed in MT solutions with 0 (Control) or 1 mM MT were treated with air and stored in the same conditions. After this period fruit were taken out from containers and placed under cold storage at 4 °C to induce CI following Megías et al. [10] conditions for 0, 3, 6, 9, 12 and 15 days + 2 days at 20 °C.

### 2.2. Postharvest Quality Parameters

Three replicates of 5 fruit were randomly selected from each treatment lot at 3 days interval during cold storage +2 days at 20 °C. Weight loss of individual zucchini fruit was calculated as percentage with respect to the weight on day 0. Firmness was determined individually as the force to achieve a 5% fruit diameter deformation in both sides and fruit firmness was expressed as N by using a Texture Analyzer (TX-XT2i, S Microsystems, Godalming, UK). Chilling injury was evaluated visually with a panel of 5 trained judges scoring superficial area affected by pitting damage and the pitting severity. Ratings were based on a 6-point hedonic scale, where the fruit surface affected was used to classify each fruit similarly to Megías et al. [31] with the following scale: 0 = no pitting, 1 = ≤5% pitting, 2 = 6–15% pitting, 3 = 16–25% pitting, 4 = 26–50% pitting, and 5 = ≥50% pitting. On the other hand, to assess the severity of pitting symptoms, the scale was 0 = no damage, 1 = very superficial damage, 2 = superficial damage, 3 = moderate damage, 4 = severe damage, 5 = very severe damage. The final CI index displayed in this manuscript was the average of both assessments.

CO_2_ and ethylene production were determined by placing individually 6 randomly selected zucchini from each treatment in a 2.2 L plastic jar hermetically sealed with a rubber stopper for 30 min. After that, 1 mL gas sample per duplicate was taken from head space and carbon dioxide was quantified by using a Shimadzu TM 14A gas chromatograph (Kyoto, Japan) equipped with thermal conductivity detector and ethylene production was evaluated with a Hewlett-Packard^TM^ 5890A gas chromatograph. Chromatographic conditions were previously described [32]. Ethylene production and respiration rate were expressed as nL g^−1^ h^−1^ and mg of CO_2_ kg^−1^ h^−1^, respectively.

Malondialdehyde (MDA) content was assayed in the peel tissue of the zucchini samples following the method of Zhang et al. [33] with modifications. The tissue sample (1.0 g) was homogenized in 10 mL 10% trichloroacetic acid solution, then centrifuged at 10,000× *g* for 10 min. 2 mL of supernatant was added to a testing tube with 6 mL of 0.6% thiobarbituric acid per duplicate and mixed vigorously. Testing tubes were held at 95 °C for 20 min. Samples were cooled rapidly, tempered at room temperature, and evaluated in a spectrophotometer (1900 UV/Vis, Shimadzu, Kyoto, Japan) where absorbance was measured at 450, 532 and 600 nm. MDA content was calculated as described by Zhang et al. [33] and expressed as μmol kg^−1^. Each assessment was repeated three times.

Electrolyte leakage (EL) was determined following Mao et al. [34] with some modifications. From each treatment, three replicates were measured, each consisting of 20 peel discs with 0.5 mm diameter obtained with a cork borer, from longitudinal 2 mm peel exocarp slices taken from opposite sides of each zucchini. After 3 rinses of 3 min each, discs were incubated in 50 mL of deionized water at room temperature with constant shaking for 30 min. Then electrical conductivity (EC) was measured (C1). Finally, samples were boiled at 100 °C for 15 min and measured to calculate total conductivity (C2). EL was expressed as percentage using the following formula: EL = (C1/C2) 100.

For chlorophyll measurement in peel tissue, six disks, each of 6.25 mm in diameter, were punched from same peel layers sliced for EL. Disks were weighed and placed immediately into 8 mL of 100% methanol. Pigments were allowed to be extracted in the dark at 30 °C for 24 h. Extract absorbance was measured using spectrophotometer (1900 UV/Vis, Shimadzu, Kyoto, Japan) at 652 and 665 nm [35].Two extractions were evaluated by replicate. Colour parameters (CIE a* and CIE b*) were individually measured on three points of the external (both sides) and internal longitudinal fruit perimeter by using a Minolta colorimeter (CRC200, Minolta Camera Co.; Kantō, Tokio, Japan) and colour was expressed as CIE hue* (180 + tan^−1^ b*/a*, if a* < 0) according the CIELab coordinates.

Total soluble solids (TSS) were determined by duplicate in the juice obtained from the pulp of mix of 5 zucchini of each replicate per lot taken with a digital refractometer Atago PR-101 (Atago Co. Ltd.; Tokyo, Japan) at 20 °C, and expressed as percentage (g 100 g^−^^1^). Also, for each replicate total acidity (TA) was determined by duplicate in the same juice by automatic titration with NaOH 0.1 N up to pH 8.1, using 1 mL of diluted juice in 25 mL distilled H_2_O, and results were expressed as the percentage of malic acid (meq. malic acid = 0.067).

### 2.3. Statistical Analysis

All data in this paper are expressed as mean ± standard error (SE). Data were subjected to analysis of variance (ANOVA). Mean comparisons were carried out using a multiple range test (Tukey’s HSD test) to find significant differences (*p* < 0.05). Different lowercase letters indicated a significant difference among treatments at the same sampling date. All analyses were performed using SPSS software package, version 22 (IBM Corp.; Armonk, NY, USA).

## 3. Results and Discussion

CI was evaluated in a previous experiment, carrying out a screen test with four different MT concentrations (0, 0.1, 0.5 and 1 mM) during 5 different immersion times (10, 30, 60, 120 and 180 min), to select the optimal MT treatment conditions (data not shown). Nine zucchini fruit were selected per MT treatment and individually evaluated after 7 d at 4 °C + 1 d at 20 °C. Visually, CI incidence was evaluated with 5 trained judges, and external quality was maintained specially for 1 mM MT dose assayed during 1 h immersion time. On the other hand, no additional benefits were observed by increasing the immersion time. Thus, immersions during 1 h with 1 mM MT were the conditions applied with or without 1-MCP in the present study.

### 3.1. Effect of Exogenous MT and 1-MCP on Weight Loss, Fruit Firmness and Cold Tolerance

**Figure 1:**
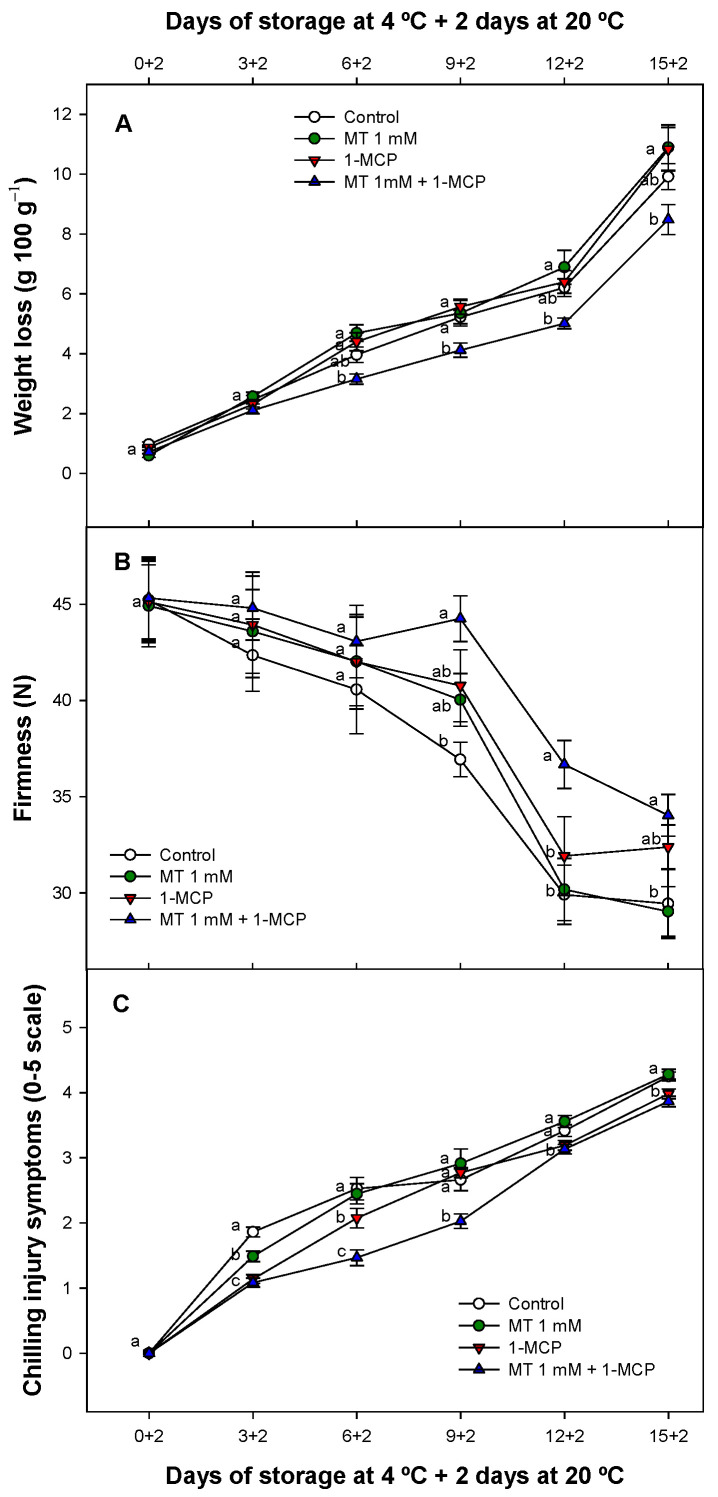
Evolution of weight losses (g 100 g^−1^) (**A**) fruit flesh firmness (N) (**B**) and chilling injury (0–5 scale) (**C**) of ‘Cronos’ zucchini fruit treated with melatonin at 1 mM (MT) or distilled water (Control) with or without 1-MCP during cold storage plus 2 days at 20 °C. Data are the mean ± SE (n = 3). Different lowercase letters show significant differences (*p* < 0.05) among treatments for each sam-pling date.

Weight loss of zucchini fruit increased throughout cold storage regardless of the treatment applied. Zucchini weight losses were not significantly (*p* ≥ 0.05) affected by MT dips and 1-MCP when applied alone. However, weight losses were significantly lower (*p* < 0.05) when combined treatments (1-MCP + MT) were evaluated during storage (Figure 1A).

In this sense the combined treatment (MT + 1-MCP) reduced weight loss (20.45%) after 6 days of cold storage plus an additional period at 20 °C as compared with the rest of the different lots evaluated. This trend was maintained until the end of the experiment.

Storage of zucchini at 4 °C plus 2 additional days at 20 °C resulted in a decrease in fruit firmness as expected (Figure 1B). However, fruit firmness levels remained higher in MT, 1-MCP and MT + 1-MCP treated fruit as compared to control fruit specially after 6 and 9 days of cold storage. On the other hand, MT and 1-MCP samples did not show significant differences (*p* ≥ 0.05) as compared to control fruit at the end of the experiment showing a similar fruit firmness level. On the contrary MT + 1-MCP samples significantly (*p* < 0.05) maintained in general a higher fruit firmness along cold storage compared with the rest of the fruit evaluated.

**Figure 2:**
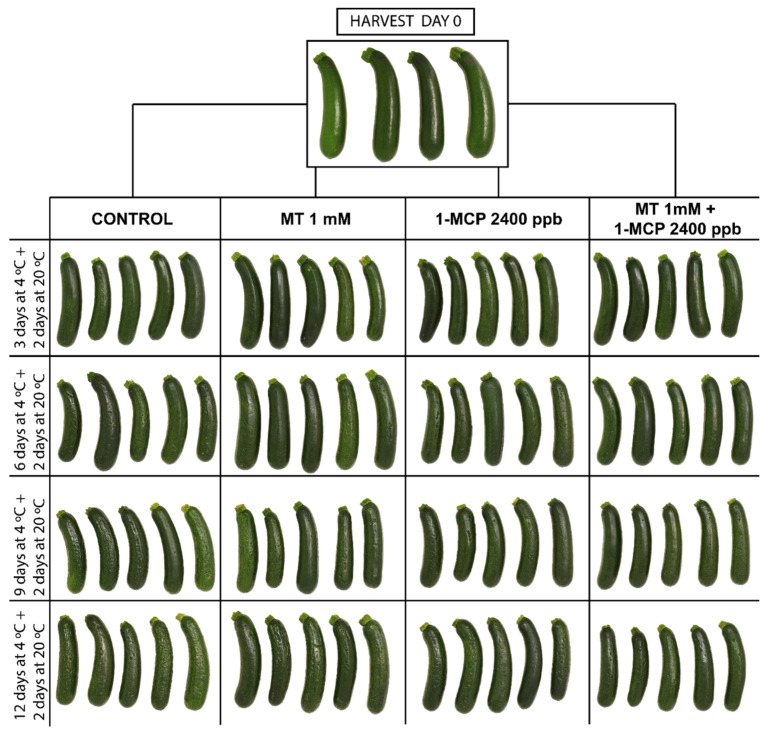
Photography displays the external visual aspect of ‘Cronos’ zucchini fruit treated with melatonin at 1 mM (MT) or distilled water (Control) with or without 1-MCP after 3, 6, 9 and 12 days of cold storage plus 2 days at 20 °C.

Zucchini fruit are very sensitive to cold storage displaying CI after 3 days of cold storage in all fruit studied (Figure 1C). The CI index was in general significantly (*p* < 0.05) higher in control fruit as compared to treated fruit with MCP and MT during storage. Although MT and 1-MCP delayed CI symptoms even after 3 and 6 days of cold storage respectively MT + 1-MCP combined treatments showed the lowest CI incidence (42.66% lower as compared to control fruit) after 6 days of refrigerated storage. According to the observations (Figure 1C) only when 1-MCP was applied combined with MT, zucchini fruit still displaying an increased chilling tolerance after 9 days of cold storage showing additional benefits when both substances were applied together. The effect of MT and 1-MCP applied alone or as combined treatment on internal disorders can be clearly observed in the photographs performed (Figure 2).

In general, in zucchini fruit, weight and fruit firmness decrease along storage specially when this fruit is stored at suboptimal temperatures mainly due to transpiration through a higher membrane permeability increased by pitting incidence. In this sense, weight loss affects cells turgor reducing fruit firmness in different fruit during storage [36,37]. For this reason, these two parameters use to be correlated between them and with CI incidence. Differences in weight loss between control and treatments were significant only when MT was applied in combination with 1-MCP. However, fruit firmness and CI were affected slightly by MT and 1-MCP alone but when were applied as a combined treatment a higher positive effect was exerted as compared to control fruit. For this reason, we inferred that 1-MCP combined with MT may have synergistic effect on these traits. 1-MCP can be effective controlling weight loss, fruit firmness and CI incidence in different non-climacteric and climacteric fruit [10,38,39,40,41]. In zucchini fruit, this trend could be cultivar dependent as it has been previously demonstrated. In fact, in Cronos cultivar, differences in weight loss were not significant when these parameters were analysed after 1-MCP treatment when stored at 4 °C [10]. These results were in consonance with our study, and we also observed an important effect for 1-MCP applied alone reducing CI. On the other hand, MT postharvest treatments have shown a strong effect delaying weight loss in some fruit [42] but a weak effect or even unaffected weight loss in different other fruit [43,44] as we observed when MT was applied alone in zucchini fruit. This slight effect also was observed on zucchini fruit firmness and CI with single MT applications. In previous studies, MT up-regulated cell wall structure-related genes [43,45]. MT also showed antioxidant activity that delayed membrane peroxidation and consequently caused a lower phenol oxidation through an effective inhibition of peroxidase (POD) and PPO activities [46,47]. Storing zucchini at suboptimal temperatures (below 7 °C) can lead to serious CI, characterized by an intense surface pitting, and sunken lesions on the skin surface, which could be caused by damage to the cell walls or cell membranes [2,12,48]. For this reason, and based in our results, we proposed that a combined effect of 1-MCP and MT delaying cell wall disassembly could allow MT antioxidant activity to control CI in a synergistic way when zucchini was treated with the combined treatment.

### 3.2. Effect of Exogenous MT and 1-MCP on Respiration Rate and Ethylene Production

**Figure 3:**
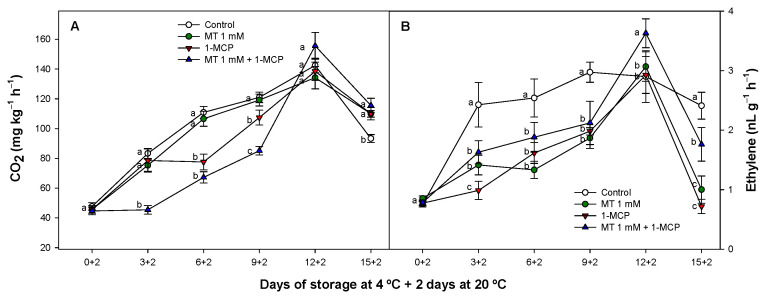
Respiration (mg CO_2_ kg^−1^ h^−1^) (**A**) and ethylene production rates (nL g^−1^ h^−1^) (**B**) of ‘Cronos’ zucchini fruit treated with melatonin at 1 mM (MT) or distilled water (Control) with or without 1-MCP during cold storage and after cold storage plus 2 days at 20 °C. Data are the mean ± SE (n = 3). Different lowercase letters show significant differences (*p* < 0.05) among treatments for each sam-pling date.

Respiration rate in zucchini fruit for all treatments tended to increase during shelf life after cold storage. However, for 1-MCP and especially for MT + 1-MCP samples respiration just increased slightly during the beginning of the study (Figure 3A) showing significant differences between treatments (*p* < 0.05).

At the end of storage CO_2_ concentrations decreased for all zucchini fruit tested but all treatments applied containing 1-MCP delayed this pattern as compared with control and MT treated fruit. On the other hand, ethylene production in zucchini fruit was low during the experiment. Control fruit significantly increased ethylene concentration (*p* < 0.05) from the beginning of the study, but the different treatments applied delayed this pattern as compared with control fruit (Figure 3A).

Respiration is a key factor involved in weight loss process showing in this study a correlation between these two parameters. Megías et al. [10] found that 1-MCP was able to delay respiration process and ethylene production in different zucchini cultivars in consonance with our results. The reduction of respiration and ethylene production has been linked to an increased cold tolerance in zucchini fruit although the impact on fruit quality during cold storage depends on cultivar [10,49]. MT regulates γ-aminobutyric acid (GABA) content in non-climacteric and climacteric fruit [17,50], stimulating GABA-shunt pathway [51]. This increase in GABA provides the cell with an immediate energy substrate that it uses to recover from stress, increasing the net energy balance in plant cells and covering the energy needs of the plant [17]. In this study, MT-treated zucchini displayed lower medium CO_2_ values but with no significant differences (*p* ≥ 0.05) as compared with the rest of treatments applied, but 1-MCP treatment alone significantly (*p* < 0.05) delayed the respiration process. On the other hand, a synergistic effect delaying the respiration process was observed when MT and 1-MCP were applied as a combined treatment probably due to an additive effect between both treatments. This additional benefit applying the combined treatment was also observed on ethylene production though MT and 1-MCP reduced cold induced ethylene production when are applied alone (Figure 3B). In this sense all the treatment delayed ethylene production until peak also delaying the decrease of ethylene at the end of storage. MT has been described as a regulator of the expression of different genes reducing ethylene production [52] and 1-MCP is a potent inhibitor of ethylene action [53]. However, the combination of treatments did not reduce ethylene production with an additional effect as compared to these treatments when applied alone. For this reason, the synergistic and beneficial effect observed on cold tolerance of zucchini (Figure 1C; Figure 2) could be explained in relation to the stimulated antioxidant balance that MT exhibits when is applied as a postharvest treatment [52] as we will describe through the following parameters (MDA and total chlorophyll content) evaluated.

### 3.3. Effect of Exogenous MT and 1-MCP on Membrane Permeability (MDA Content and EL)

**Figure 4:**
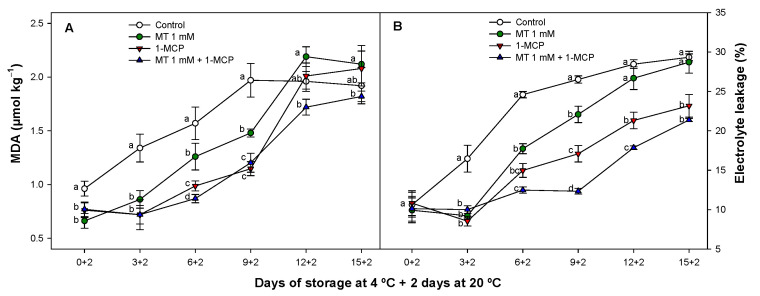
Evolution of malondialdehyde (MDA) content (µmol kg^−1^) (**A**) an electron leakage (EL) (%) (**B**) of ‘Cronos’ zucchini fruit treated with melatonin at 1 mM (MT) or distilled water (Control) with or without 1-MCP during cold storage and after cold storage plus 2 days at 20 °C. Data are the mean ± SE (n = 3). Different lowercase letters show significant differences (*p* < 0.05) among treatments for each sam-pling date.

According to the results (Figure 4A) although all treatments showed a delay in the MDA accumulation, this parameter was significantly lower (*p* < 0.05) in the cases of 1-MCP and MT + 1-MCP compared to that of control fruit.

MDA in zucchini fruit treated with MT, 1-MCP or MT + 1-MCP was reduced by 19.7, 33.6 and 44.6% on the 6th day of storage respectively, as compared to control. This delay was observed only for MT + 1-MCP after 12 days of cold storage plus 2 days at 20 °C.

MT and 1-MCP alone or as a combined treatment (MT + 1-MCP) significantly (*p* < 0.05) delayed EL evolution (Figure 4B). However, a lower EL was observed especially when these compounds were applied as a combined treatment since the lowest EL was observed during storage when MT + 1-MCP were applied. On the contrary MT applied alone was the treatment with a weaker effect on EL. MDA reflects the lipid peroxidation of plasma membranes which directly affects the structural integrity of vegetal tissues [54]. Previous works have suggested that 1-MCP affects the activities of antioxidant enzymes [55,56] as well as MT treatments on different fruit [57,58] reducing MDA content and the impact on EL in consonance with our results. For this reason, the similar effect observed on these parameters after applying 1-MCP or MT alone, could be the reason why the reduction in MDA content and EL evolution was greater when both substances were applied together displaying an additive cold tolerance effect.

### 3.4. Effect of Exogenous MT and 1-MCP on Chlorophyll Content and External Colour

**Figure 5:**
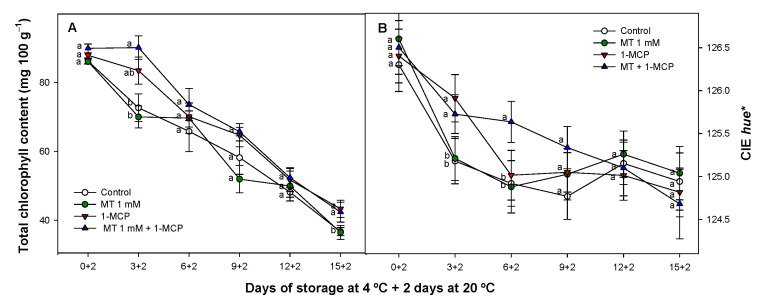
Evolution of total chlorophyll content (mg 100 g^−1^) (**A**) and CIE hue* (**B**) of ‘Cronos’ zucchini fruit treated with melatonin at 1 mM (MT) or distilled water (Control) with or without 1-MCP during cold storage and after cold storage plus 2 days at 20 °C. Data are the mean ± SE (n = 3). Different lowercase letters show significant differences (*p* < 0.05) among treatments for each sam-pling date.

Chlorophyll content in treated and untreated samples showed a decreased pattern for all fruit tested as it was expected (Figure 5A).

Interestingly there was a clear effect provided by 1-MCP alone or combined with MT showing a positive effect on the maintenance of this parameter after 3 days of storage (83.44 ± 3.93 and 90.11 ± 3.41 mg 100 g^−1^ fw respectively). These values were significant higher (*p* < 0.05) than observed for MT and control fruit (70.00 ± 3.17 and 72.64 ± 4.01 mg 100 g^−1^ fw respectively). This positive effect was in general maintained along the whole experiment. On the other hand, when CIE hue* slightly decreased during storage in all zucchini fruit tested, 1-MCP and MT + 1-MCP lots maintained significant differences (*p* < 0.05) as compared with the rest of treatments applied (Figure 5B) though these differences were reduced along the experiment. However, when MT and 1-MCP were applied as a combined treatment these differences were maintained delaying the evolution of this parameter for longer time than when these substances were applied alone.

Green colour in zucchini is determined by chlorophyll content and storage impact on pigment degradation is correlated with a reduced CIE hue* in Cronos cultivar [59]. These authors also observed that zucchini fruit senescence is accompanied by decrease of chlorophyll pigments and CIE hue* during storage in Cronos cultivar mainly due to loss of cell wall integrity, reducing firmness and contributing to chlorophyll pigment degradation [59]. In this sense 1-MCP treatments have been shown to maintain tissue firmness and chlorophyll content delaying senescence in climacteric and non-climacteric fruit [25,53]. On the other hand, chlorophyll pigments are also affected during postharvest as the main targets of ROS-linked damage since ROS detoxification systems decrease during plant senescence and other different stresses [60]. MT treatments have been shown to delay tissue degreening maintaining chlorophyll content in different other MT-treated vegetal products as broccoli, mango, or cucumber [61,62,63]. In our study, 1-MCP treatments maintained these parameters, but MT treatment did not affect both of them. For this reason, the synergistic effect observed when treatments were applied combined, could be due to a better antioxidant MT performance mediated by an improved cell homeostasis since 1-MCP when applied alone, also showed a better control over chlorophyll content, MDA and EL than observed for MT-treated fruit for all these parameters.

### 3.5. Effect of Exogenous MT and 1-MCP on TSS and TA

**Figure 6:**
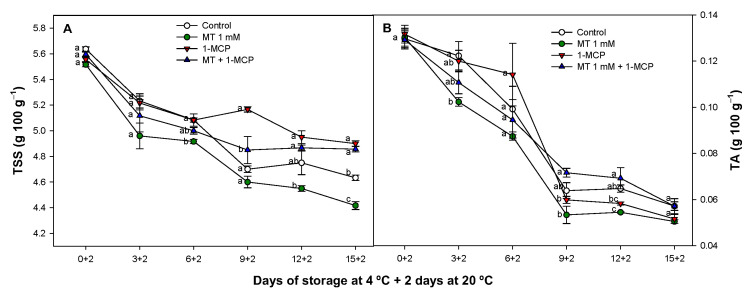
Evolution of total soluble solids (g 100 g^−1^) (**A**) and titratable acitidity (g 100 g^−1^) (**B**) of ‘Cronos’ zucchini fruit treated with melatonin at 1 mM (MT) or distilled water (Control) with or without 1-MCP during cold storage and after cold storage plus 2 days at 20 °C. Data are the mean ± SE (n = 3). Different lowercase letters show significant differences (*p* < 0.05) among treatments for each sam-pling date.

TSS in zucchini fruit is shown in Figure 6A exhibiting a decrease in all treatments.

TSS content in 1-MCP and MT + 1-MCP groups was significantly higher (*p* < 0.05) as compared to control fruit after 9 days of 4 °C storage. On the other hand, MT fruit displayed the lowest values during the storage period decreasing to 4.41 ± 0.03 g 100 g^−1^ at the end of the storage.

Similarly to TSS, TA in all the groups evaluated decreased during storage conditions and when MT was applied alone TA levels were lower as compared to the rest of fruit groups studied (Figure 6B). 1-MCP alone retained initial TA levels during 6 days of storage but as compared to control fruit no significant differences (*p* ≥ 0.05) were observed. However, fruit treated with MT + 1-MCP exhibited higher TA levels than the rest of treatments after 9 days of cold storage plus an additional period of 2 days at 20 °C.

TSS content is an important attribute which reflects the sugar concentration in cells which increases through the conversion of starch to sugar. However, in this study and in consonance with previous studies on zucchini fruit it seems that at 20 °C, the sugar consumption is faster than its accumulation leading to senescence [33,64]. On the other hand, is well documented the decreased TA level in zucchini and other different fruits by the use of organic acids as respiration substrates during ripening process [64,65]. Previous reports have revealed the effect of 1-MCP on delaying ripening processes as decreasing the respiration rate in zucchini [10] and maintaining TSS and TA content through this mechanism in different fruit species [53,65]. There are no previous studies of the effect of MT treatments on zucchini fruit though in a recent review MT was described as a substance capable of inducing cold tolerance by the accumulation of sugar and organic acids in different fruit species [66]. However, MT treatments did not increase TSS or TA in zucchini probably due to the similar respiration pattern in MT-treated fruit than observed for control fruit (Figure 3A). For this reason and based in our results, the combined treatment (MT + 1-MCP) though did not increase TSS to a higher concentration than that observed for 1-MCP alone, a synergistic effect maintaining higher TA levels was displayed when both treatments were applied together showing the highest levels of this parameter. We propose that this additional benefit could be due to a reduction in the ripening process and respiration caused by the 1-MCP but also to an increase in organic acids as have been observed in different fruit species treated with MT [66]. In this sense, a higher solute concentration is a positive factor for maintaining the protoplasm osmoregulation enhancing the cold tolerance. Higher levels in TSS and TA are directly related with a higher cell homeostasis and higher contents in sugars and organic acids as ascorbic or citric acid may also contribute to a reduced CI impact [66,67,68].

## 4. Conclusions

The present study confirmed that melatonin at 1 mM concentration as a postharvest dip treatment when combined with 1-MCP can extend the storage life of zucchini by reducing respiratory metabolism and maintaining fruit firmness reducing weight loss. The reduced metabolism observed by 1-MCP, combined with a melatonin treatment with an also displayed antioxidant effect observed maintaining chlorophyll content or reducing MDA accumulation were crucial factors on cell membrane integrity increasing zucchini cold tolerance. Also, an increased solute concentration observed in zucchini exposed to the combined treatment, could determine zucchini cold tolerance during storage at suboptimal temperatures. In this sense, results suggest that application of a combined treatment based on melatonin and 1-MCP could be a promising tool to increase storability of this fruit.

## Data Availability

Data are contained within the article.
